# The sequence of structural, functional and cognitive changes in multiple sclerosis

**DOI:** 10.1016/j.nicl.2020.102550

**Published:** 2020-12-24

**Authors:** Iris Dekker, Menno M. Schoonheim, Vikram Venkatraghavan, Anand J.C. Eijlers, Iman Brouwer, Esther E. Bron, Stefan Klein, Mike P. Wattjes, Alle Meije Wink, Jeroen J.G. Geurts, Bernard M.J. Uitdehaag, Neil P. Oxtoby, Daniel C. Alexander, Hugo Vrenken, Joep Killestein, Frederik Barkhof, Viktor Wottschel

**Affiliations:** a Amsterdam UMC, Location VUmc, Departments of Radiology and Nuclear Medicine, MS Center Amsterdam, Amsterdam Neuroscience, De Boelelaan 1117, Amsterdam, The Netherlands; b Neurology, MS Center Amsterdam, Amsterdam Neuroscience, De Boelelaan 1117, Amsterdam, The Netherlands; c Anatomy and Neurosciences, MS Center Amsterdam, Amsterdam Neuroscience, De Boelelaan 1117, Amsterdam, The Netherlands; d Biomedical Imaging Group Rotterdam, Departments of Medical Informatics and Radiology & Nuclear Medicine, Erasmus Medical Center, Rotterdam, The Netherlands; e Dept. of Diagnostic and Interventional Neuroradiology, Hannover Medical School, Hannover, Germany; f Centre for Medical Image Computing, Department of Computer Science, UCL, London, UK; g Institute of Neurology, UCL, London, UK

**Keywords:** Multiple sclerosis, Disease progression, Disability, Cognition, MRI, Event-based modelling

## Abstract

**Background:**

As disease progression remains poorly understood in multiple sclerosis (MS), we aim to investigate the sequence in which different disease milestones occur using a novel data-driven approach.

**Methods:**

We analysed a cohort of 295 relapse-onset MS patients and 96 healthy controls, and considered 28 features, capturing information on T2-lesion load, regional brain and spinal cord volumes, resting-state functional centrality (“hubness”), microstructural tissue integrity of major white matter (WM) tracts and performance on multiple cognitive tests. We used a discriminative event-based model to estimate the sequence of biomarker abnormality in MS progression in general, as well as specific models for worsening physical disability and cognitive impairment.

**Results:**

We demonstrated that grey matter (GM) atrophy of the cerebellum, thalamus, and changes in corticospinal tracts are early events in MS pathology, whereas other WM tracts as well as the cognitive domains of working memory, attention, and executive function are consistently late events. The models for disability and cognition show early functional changes of the default-mode network and earlier changes in spinal cord volume compared to the general MS population. Overall, GM atrophy seems crucial due to its early involvement in the disease course, whereas WM tract integrity appears to be affected relatively late despite the early onset of WM lesions.

**Conclusion:**

Data-driven modelling revealed the relative occurrence of both imaging and non-imaging events as MS progresses, providing insights into disease propagation mechanisms, and allowing fine-grained staging of patients for monitoring purposes

## 1 Introduction

Multiple sclerosis (MS) is a chronic inflammatory, demyelinating and neurodegenerative disease of the central nervous system (CNS) (Longo et al., 2018) frequently leading to physical disability and cognitive decline (Compston and Coles, 2008). The underlying pathological processes result in tissue damage, leaving behind demyelinating lesions and white (WM) and grey matter (GM) atrophy that can be visualised and quantified by brain and spinal cord imaging (Dekker and Wattjes, 2017). Alterations in structural and functional networks of the brain also have clear clinical relevance (Filippi et al., 2014). Usually considered in isolation, various studies have considered these features of MS. However, the sequence in which these changes occur remains unclear, in part due to scarcity of longitudinal data.

Event-based modelling (EBM) is a probabilistic data-driven approach to study disease progression that uses cross-sectional data to estimate the temporal sequence of events and subsequently stage patients within this sequence (Fonteijn et al., 2012, Young et al., 2014). This type of model has been applied in Alzheimer’s disease (Fonteijn et al., 2012, Young et al., 2014, Oxtoby et al., 2018), Huntington’s disease (Fonteijn et al., 2012, Wijeratne et al., 2018), and a recent EBM study in MS patients provided insights into the sequence of GM atrophy, but did not include features derived from other modalities (Eshaghi et al., 2018).

In the present study we go beyond the aspect of atrophy in MS and consider a broader set of structural, functional, and cognitive outcomes. We explored measures quantifying demyelination (focal WM lesions) (Compston and Coles, 2008), neurodegeneration (GM atrophy) (Bergsland et al., 2012), microstructural changes of WM tracts (fractional anisotropy) (Huang et al., 2018), and functional centrality of key brain networks (Filippi et al., 2014, Schoonheim et al., 2014) using a discriminative EBM (dEBM), which is more accurate and computationally efficient than the original EBM implementation (Venkatraghavan et al., 2019). The imaging biomarkers were supplemented with measures of cognitive performance (Schoonheim et al., 2012). Our multimodal dEBM could improve the interpretation of studies using single biomarkers, provide useful insights into disease propagation mechanisms, and aid in fine-grained staging and precise monitoring of patients. Therefore, the primary aim was to build a model that reflects a sequence of events in disease evolution in MS patients with a relapse onset. The secondary aim was to explore the event sequence for patients in relation to worsening physical and cognitive burden separately, because underlying disease processes could be different.

## 2 Methods

### 2.1 Participants

In this retrospective analysis study, we included data from the Amsterdam MS cohort based on the availability of multimodal data, resulting in the inclusion of 96 healthy controls (HC) and 295 patients with relapse-onset MS (ROMS) according to the 2011 revisions of the McDonald criteria (Polman et al., 2011). Patients with a primary progressive disease onset have been excluded.

The institutional ethics review board of the VU University Medical Center approved the protocol and written informed consent was obtained from all participants prior to inclusion.

### 2.2 Clinical assessments

The Expanded Disability Status Scale (EDSS) score was assessed in all patients and was used to classify patients into three groups according to having minimal (EDSS 0.0 – 2.5), moderate (EDSS 3.0 – 3.5) or severe disability (EDSS ≥ 4.0) as defined in (Kurtzke, 1983). Cognitive performance was assessed in all patients and HCs using an expanded Brief Repeatable Battery of Neuropsychological tests (Rao, 1990) with different cognitive domains tested, as described previously (Schoonheim et al., 2012). Raw test scores were corrected for the confounding effects of sex, age and education trends seen in the HCs (Amato et al., 2006). Cognitive domain-specific z-scores were calculated using the mean and standard deviation (SD) of the HCs. Patients were sub-divided into three cognitive-performance groups according to the z-scores obtained from the neuropsychological tests. Patients with z≤-2 on at least 2 out of 7 cognitive domains of the neuropsychological tests were labelled as cognitively impaired (CI), patients with z≤-1.5 on at least 2 cognitive domains but not fulfilling CI criteria were classified as mildly cognitively impaired (MCI) and the remaining patients were classified as cognitively preserved (CP) (Schoonheim et al., 2015). Level of education was measured using a scale ranging from 1 (unfinished primary school) to 7 (a university degree or higher) (Verhage and Van Der Werff, 1964).

### 2.3 Magnetic resonance imaging

A 3 Tesla whole-body MR system was used to scan all participants (GE Signa HDxt, Milwaukee, WI) using an 8-channel phased-array head coil. The scan protocol included a 3D T1-weighted fast spoiled gradient-echo sequence for volume measures (TR: 7.8 ms, TE: 3 ms, 240 × 240 mm^2^ field of view (FOV), 176 sagittal slices of 1 mm thickness, 0.94 × 0.94 mm^2^ in-plane resolution), a 3D fluid-attenuated inversion recovery (FLAIR) sequence for lesion detection (TR: 8000 ms, TE: 125 ms, TI: 2350 ms, 250 × 250 mm^2^ FOV, 132 sagittal slices of 1.2 mm thickness, 0.98 × 0.98 mm^2^ in-plane resolution), a diffusion-weighted imaging (DWI) sequence to detect microstructural changes in WM tracts (TR: 13 s, TE: 86 ms, 2.4 mm contiguous axial slices, 2.0 × 2.0 mm^2^ in-plane resolution, 30 volumes with b-value = 900 s/mm^2^, 5 volumes with b-value = 0 s/mm^2^), and a whole-brain resting-state fMRI sequence to measure eigenvector centrality (functional centrality; (200 volumes, TR: 2200 ms, TE: 35 ms, 3 mm contiguous axial slices covering the entire brain, 3.3 × 3.3 mm^2^ in‐plane resolution)). FLAIR images were generally only acquired for patient, not HCs. More details on the protocol can be found in a previous report on this cohort (Eijlers et al., 2018).

FLAIR images were used to segment WM lesions in MS patients using a k-Nearest-Neighbours approach with tissue type priors (kNN-TTP) (Steenwijk et al., 2013). Lesion maps were registered to 3D T1-weighted images and filled using a validated patch-based approach (Prados et al., 2016).

Brain parcellation of cortical and subcortical regions was obtained using geodesic information flows (GIF) (Cardoso et al., 2015) on the 3D T1-weighted MRI scans, a method that has been used previously in applications of MS (Eshaghi et al., 2018, Pardini et al., 2016), including a predecessor study on EBM-based atrophy progression (Eshaghi et al., 2018), and other neurological disorders (Ingala et al., 2020), as well as a pre-processing tool for segmenting WM hyperintensities (Sudre et al., 2017). GIF is an atlas-propagation-based method that registers T1 scans of 160 subjects with manually delineated brain structures to each target scan, then identifies the closest local matches and uses those matches for segmentation. The atlas segmentations are based on the Desikan-Killiany-Tourville protocol, which was designed to improve accuracy and consistency of brain labels compared to the classic Desikan-Killiany atlas database (Klein and Tourville, 2012). To further quantify regional lesion loads, the white matter was initially divided into 10 concentric bands between the ependyma of the ventricles and the pial surface based on normalized subject-specific distance maps derived from Laplace equation isolines (Pardini et al., 2016, Sudre et al., 2018). The bands were then grouped as inner (band 1–2), intermediate deep (band 3–8), and outer bands (band 9–10) to in order to obtain a data-driven approximation of the stratification used in (pre-)clinic. Infratentorial lesions were subsequently discarded because they were only present in a small subset of patients.

Spinal cord atrophy was quantified as mean upper cervical cord area (MUCCA) using SCT-PropSeg (De Leener et al., 2014). Analyses were performed on the 3D T1-weighted images of the brain, which cover a sufficient length of the cervical spinal cord. We measured over a length of 30 mm along the central canal, starting at the top of the second cervical vertebra, C2. MUCCA measurements on brain images have been shown to be as reproducible as those performed on dedicated spinal cord MRI (Lukas et al., 2018, Liu, 2016).

Functional MRI processing steps for obtaining eigenvector centrality maps (ECM) have been published previously (Eijlers et al., 2017). The MELODIC pipeline (part of FSL (Smith et al., 2004), using standard settings) was used to process resting-state fMRI images, followed by nonlinear registration to Montreal Neurological Institute standard space, and resampling to a resolution of 4 mm isotropic. The MELODIC outcomes were further processed using fastECM (Wink et al., 2012) to estimate voxel-wise eigenvector centrality as a network measure of functional hubness (brain function) in the default-mode network (DMN), basal ganglia and sensorimotor network.

DWI scans were pre-processed using FSL5, including motion- and eddy current correction on images and gradient vectors, followed by diffusion tensor fitting for diffusion tensor imaging (DTI). The resulting fractional anisotropy (FA) maps were then fed into the tract-based spatial statistics (TBSS) pipeline (Meijer, 2018), after which the skeleton was masked using the JHU white-matter tractography atlas from FSL to define WM tracts (Mori, 2005).

There was only a minor amount of motion artefacts present in the advances imaging sequences, and we did not observe any difference in artefact severity between groups.

### 2.4 Discriminative event-based model

The EBM uses cross-sectional data to estimate the ordered sequence of cumulative abnormality in a disease, together with uncertainty in the ordering. Here, we used the discriminative EBM (dEBM; https://github.com/EuroPOND/pyebm) described previously as it has been shown to be more accurate and computationally efficient compared to other EBM implementations (Venkatraghavan et al., 2019, Venkatraghavan et al., 2017). The dEBM estimates the probability for each biomarker being normal or abnormal using a Gaussian mixture model (GMM) based on data from a disease and a reference population. Based on the probability distributions of the biomarkers in the two groups, an individual sequence of biomarker abnormality is calculated for each patient. Finally, these individual sequences are combined statistically to give an ordering for the whole population (Venkatraghavan et al., 2019). The uncertainty of this ordering is estimated by bootstrapping, i.e. repeating the experiment with random subsets of subjects. Subjects can be staged within the event sequence by identifying the events that have already become abnormal for each individual subject (Young et al., 2014).

### 2.5 Selected biomarkers

We included multimodal biomarkers with relevance in MS whilst limiting the overall number of features in the model to allow for better interpretability of results and faster computation. The following 28 MS-related biomarkers were considered (before statistical post-selection as described below):

*GM volumes* of the thalamus, hippocampus, basal ganglia (without thalamus and hippocampus), cerebellar GM, cingulate, frontal lobe, insula, occipital lobe, parietal lobe and temporal lobe. These regions cover the entire brain to allow for a rough estimate of the general atrophy sequence.

*MUCCA* was included for all subjects as an indicator of spinal cord volume.

*T2-hyperintense lesion loads* on FLAIR images were considered only for patients and split according to the inner (i.e., periventricular), outer (i.e., juxtacortical) and intermediate deep WM bands in order to obtain a data-driven approximation of the stratification used in (pre-)clinic.

*Functional centrality* in the default mode network (DMN), sensorimotor cortex network and basal ganglia network; the voxelwise ECM-measures were averaged within the respective anatomical regions. The selected networks are linked to MS progression in the domains cognition (Eijlers et al., 2017), fatigue (Finke, 2015) and clinical recovery (Mezzapesa et al., 2008).

*Microstructural changes of WM tracts measured by fractional anisotropy (FA)* in 3 major WM tracts related to cognition (anterior thalamic radiation and cingulum (Eijlers, 2018, Koenig, 2015) and motor function (corticospinal tract (Reich et al., 2007) and all other WM tracts combined (forceps minor, forceps major, inferior fronto-occipital fasciculus, inferior longitudinal fasciculus, superior longitudinal fasciculus, and uncinate fasciculus); the voxelwise FA-measures were averaged within the respective anatomical regions.

*Cognitive function* by cognitive domain: executive function, verbal memory, information processing, verbal fluency, visuospatial, working memory and attention.

### 2.6 Statistics

Normality of data was checked by visual inspection of histograms combined with Kolmogorov-Smirnov testing. Parametric (independent-samples *t*-test) and non-parametric (Mann-Whitney *U* test and chi-square test) tests were used to compare groups () for demographic, clinical and imaging characteristics (Table 1, Table 2, Table 3). All measures, except lesions, which could only be obtained in patients, were corrected for the confounding effects of age, sex and education seen in HCs using one linear regression model per biomarker. The residuals of these fits were then transformed into z-scores using the mean and SD from HCs.

**Table 1:** Clinical and imaging measures, patients and healthy controls.

**Clinical measures**		**Patients (n = 295)**	**Healthy controls (n = 96)**	***p*-value**
	Age [years] *	47.0 ± 10.7	45.9 ± 10.4	0.37 ^a^
	Sex [female, %] ^**^	211 (71.5)	56 (58.3)	**0.016^b^**
	Education level ^***^	5 (4 – 6)	6 (4 – 7)	**0.017^c^**
	Symptom duration [years] *	12.6 ± 1.6	N/A	–
	DMT ever used [%]^**^	173 (58.6)	N/A	–
	EDSS ^***^	3.0 (2.0 – 4.0)	N/A	–
	CP/MCI/CI [%] ^**^	25.4 / 17.6 / 56.9	N/A	–
	RRMS/SPMS	243/52	N/A	–
	Information processing speed [z-score] *	−1.12 (1.4)	0.0 (1.0)	**<0.001^a^**
	Executive functioning [z-score] *	−0.95 (1.7)	0.0 (0.8)	**<0.001^a^**
	Working memory [z-score] *	−1.02 (1.5)	0.0 (0.9)	**<0.001^a^**
	Verbal memory [z-score] *	−0.48 (1.2)	0.0 (0.9)	**<0.001^a^**
	Verbal fluency [z-score] *	−0.44 (1.1)	0.0 (1.0)	**<0.001^a^**
	Visuospatial memory [z-score] *	−0.61 (1.2)	0.0 (0.9)	**<0.001^a^**
	Attention [z-score] *	−0.65 (1.1)	0.0 (0.7)	**<0.001^a^**
**MRI measures**				
T2-hyperintense lesion loads [mL]*				
	Total T2-hyperintense lesion load	14.2 (12.7)	N/A	–
	Inner lesions	4.3 (3.3)	N/A	–
	Deep lesions	6.5 (6.9)	N/A	–
	Outer lesions	3.4 (3.5)	N/A	–
	Infratentorial	0.02 (0.04)	N/A	–
*Brain and spinal cord volumes [mL]* *				
	Total brain volume	1135.3 (110.3)	1181.8 (128.8)	**<0.001 ^a^**
	Basal Ganglia	34.3 (3.5)	36.7 (4.1)	**<0.001 ^a^**
	Hippocampus	7.6 (0.7)	8.0 (0.8)	**<0.001 ^a^**
	Thalamus	10.1 (1.0)	11.7 (1.4)	**<0.001 ^a^**
	Cingulate	27.7 (3.3)	29.0 (3.6)	**0.002 ^a^**
	Frontal lobe	179.92 (18.8)	185.1 (20.8)	0.224 **^a^**
	Insula	10.7 (1.2)	11.3 (1.4)	**<0.001 ^a^**
	Occipital lobe	66.6 (8.0)	70.3 (8.2)	**<0.001 ^a^**
	Parietal lobe	91.1 (10.2)	95.0 (9.6)	**0.002 ^a^**
	Temporal lobe	128.0 (13.4)	132.8 (15.0)	**0.006 ^a^**
	Cerebellar grey matter	94.6 (9.5)	99.0 (10.1)	**0.003 ^a^**
	MUCCA	64.7 (7.8)	68.6 (5.7)	**<0.001 ^a^**
*Functional hubness (EC [z-scores])*				
	Basal ganglia network	−0.128 (0.23)	0 (0.26)	0.371 **^a^**
	Default mode network	0.009 (0.21)	0 (0.22)	0.755 **^a^**
	Sensorimotor cortex network	−0.046 (0.22)	0 (0.22)	0.234 **^a^**
*White matter tract integrity (FA [0–1])*				
	Anterior thalamic radiation	0.453 (0.035)	0.479 (0.027)	**<0.001 ^a^**
	Corticospinal tract	0.653 (0.028)	0.668 (0.028)	**<0.001 ^a^**
	Cingulum	0.564 (0.047)	0.598 (0.041)	**<0.001 ^a^**
	Other WM tracts	0.525 (0.035)	0.561 (0.026)	**<0.001 ^a^**

* Mean (standard deviation), ^**^ number (percentage), ^***^ median (IQR). ^a^ Independent-samples *t*-test, ^b^ chi-square test, ^c^ Mann-Whitney *U* test. CI: cognitively impaired; CP: cognitively preserved; DMT: disease modifying treatment; EC: eigenvector centrality; EDSS: expanded disability status scale; FA: fractional anisotropy; MCI: mild cognitively impaired; MUCCA: mean upper cervical cord area; N/A: not applicable.

Table 1 shows the clinical and imaging measures of patients and healthy controls. The *p*-values of the MRI measures are based on the z-scored comparisons. Biomarkers with a *p*-value < 0.1 were included in the model.

**Table 2:** Demographics high vs low EDSS.

**Clinical measures**		**Total(n = 222)**	**EDSS ≤ 2.5(n = 120)**	**EDSS ≥ 4.0(n = 102)**	***p*-value**
Age [years]*		46.5 (10.6)	41.0 (8.5)	53.1 (8.9)	**<0.001^a^**
Sex [female, %]^**^		160 (72.1)	87 (72.5)	73 (71.6)	0.88^b^
Education level [median, IQR]^***^		5 (4 – 6)	5 (4 – 6)	4 (3 – 6)	**0.001^c^**
Symptom duration [years]*		14.4 (8.4)	10.6 (5.7)	18.8 (8.8)	**<0.001^a^**
DMT used^**^		126 (56.8)	67 (55.8)	59 (57.8)	0.76^b^
EDSS^***^		2.5 (2.0 – 4.5)	2.0 (1.5 – 2.5)	5.0 (4.0 – 6.0)	**<0.001^c^**
CP/MCI/CI [%]^***^		53.6 / 17.6 / 28.8	68.3 / 18.3 / 13.3	36.3 / 16.7 / 47.1	**<0.001^b^**
RRMS/SPMS		176 / 46	119 / 1	57 / 45	**<0.001^b^**
Information processing speed [z-score] *		−1.18 (1.4)	−0.67 (1.2)	−1.80 (1.4)	**<0.001^a^**
Executive functioning [z-score] *		−1.04 (1.8)	−0.50 (1.0)	−1.71 (2.4)	**<0.001^a^**
Working memory [z-score] *		−1.03 (1.4)	−0.51 (1.0)	−1.69 (1.6)	**<0.001^a^**
Verbal memory [z-score] *		−0.45 (1.1)	−0.25 (1.0)	−0.70 (1.2)	**0.002^a^**
Verbal fluency [z-score] *		−0.52 (1.1)	−0.22 (1.0)	−0.86 (1.0)	**<0.001^a^**
Visuospatial memory [z-score] *		−0.63 (1.2)	−0.28 (1.1)	−1.04 (1.2)	**<0.001^a^**
Attention [z-score] *		−0.66 (1.1)	−0.39 (0.9)	−0.98 (1.3)	**<0.001^a^**
**MRI measures**					
T2-hyperintense lesion loads [mL]*					
	Total T2-hyperintense lesion load	14.7 (13.2)	11.2 (9.0)	18.9 (15.9)	**<0.001^a^**
	Inner lesions	4.3 (3.3)	3.6 (2.5)	5.2 (3.8)	**<0.001^a^**
	Deep lesions	6.9 (7.4)	5.1 (4.9)	9.1 (9.2)	**<0.001^a^**
	Outer lesions	3.5 (3.4)	2.6 (2.3)	4.6 (4.1)	**<0.001^a^**
	Infratentorial	0.01 (0.04)	0.01 (0.03)	0.02 (0.04)	0.31 ^a^
*Brain and spinal cord volumes [mL]* *					
	Total brain volume	1133.0 (111.3)	1153.1 (109.4)	1109.4 (109.4)	**0.003^a^**
	Basal Ganglia	34.2 (3.6)	34.9 (3.6)	33.3 (3.5)	**<0.001^a^**
	Hippocampus	7.6 (0.7)	7.7 (0.8)	7.4 (0.7)	**0.011 ^a^**
	Thalamus	10.1 (1.5)	10.6 (1.3)	9.4 (1.3)	**<0.001^a^**
	Cingulate	27.7 (3.3)	28.1 (3.2)	27.2 (3.4)	**0.04 ^a^**
	Frontal lobe	180.1 (19.0)	185.4 (19.6)	173.9 (16.2)	**<0.001^a^**
	Insula	10.7 (1.3)	11.0 (1.3)	10.3 (1.2)	**<0.001^a^**
	Occipital lobe	66.3 (8.0)	68.3 (7.8)	64.0 (7.7)	**<0.001^a^**
	Parietal lobe	91.1 (10.2)	94.0 (10.2)	87.8 (9.3)	**<0.001^a^**
	Temporal lobe	127.7 (13.5)	130.7 (13.9)	124.1 (12.1)	**<0.001^a^**
	Cerebellar grey matter	94.3 (9.7)	96.6 (9.3)	91.6 (9.5)	**<0.001^a^**
	MUCCA	64.5 (8.3)	65.6 (8.3)	63.2 (8.2)	0.07 ^a^
*Functional hubness (EC [z-scores])*					
	Basal ganglia network	−0.016 (0.24)	−0.067 (0.235)	0.044 (0.224)	**<0.001^a^**
	Default mode network	−0.004 (0.21)	−0.035 (0.221)	0.033 (0.200)	**0.017^a^**
	Sensorimotor cortex network	−0.039 (0.22)	−0.021 (0.216)	−0.058 (0.216)	0.206^a^
*White matter tract integrity (FA [0*–*1])*					
	Anterior thalamic radiation	0.45 (0.03)	0.47 (0.02)	0.44 (0.04)	**<0.001^a^**
	Corticospinal tract	0.65 (0.03)	0.66 (0.02)	0.64 (0.03)	**<0.001^a^**
	Cingulum	0.56 (0.05)	0.58 (0.04)	0.55 (0.05)	**<0.001^a^**
	Other WM tracts	0.53 (0.04)	0.54 (0.03)	0.51 (0.04)	**<0.001^a^**

* Mean (standard deviation), ^**^ number (percentage), ^***^ median (IQR). ^a^ Independent-samples *t*-test, ^b^ chi-square test, ^c^ Mann-Whitney *U* test.

CI: cognitively impaired; CP: cognitively preserved; DMT: disease modifying treatment, EDSS: expanded disability status scale, IQR: interquartile range, MCI: mild cognitively impaired.

Table 2 shows the clinical measures of the patient group split into low and higher EDSS.

**Table 3:** Demographics cognitive preserved vs cognitive impaired

**Clinical measures**		**Total(n = 243)**	**Cognitive preserved (n = 168)**	**Cognitive impaired (n = 75)**	***p*-value**
Age [years]*		47.1 (10.6)	45.7 (10.3)	50.4 (10.5)	**0.001^a^**
Sex [female, %]^**^		174 (71.6)	125 (74.4)	49 (65.3)	0.15^b^
Education level [median, IQR]^***^		5 (4 – 6)	6 (4 – 6)	4 (3 – 6)	**<0.001^c^**
Symptom duration [years]*		14.6 (8.6)	13.3 (7.7)	17.6 (9.6)	**<0.001^a^**
DMT used^**^		141 (58.0)	96 (57.1)	45 (60.0)	0.68^b^
EDSS^***^		3.0 (2.0 – 4.0)	3.0 (2.0 – 3.5)	4.0 (3.0 – 6.0)	**<0.001^c^**
RRMS/SPMS		197 / 46	148 / 20	49 / 26	**<0.001^b^**
Information processing speed [z-score] *		−1.01 (1.4)	−0.35 (1.0)	−2.52 (1.2)	**<0.001^a^**
Executive functioning [z-score] *		−0.95 (1.8)	−0.19 (0.8)	−2.73 (2.3)	**<0.001^a^**
Working memory [z-score] *		−0.99 (1.6)	−0.39 (0.8)	−2.40 (2.0)	**<0.001^a^**
Verbal memory [z-score] *		−0.44 (1.2)	0.02 (0.9)	−0.15 (1.1)	**<0.001^a^**
Verbal fluency [z-score] *		−0.38 (1.1)	−0.04 (1.0)	−1.2 (1.0)	**<0.001^a^**
Visuospatial memory [z-score] *		−0.56 (1.2)	−0.14 (1.0)	−1.49 (1.1)	**<0.001^a^**
Attention [z-score] *		−0.60 (1.2)	−0.22 (0.7)	−1.48 (1.6)	**<0.001^a^**
**MRI measures**					
T2-hyperintense lesion loads [mL]*					
	Total T2-hyperintense lesion load	13.7 (12.8)	10.3 (8.4)	21.4 (17.0)	**<0.001^a^**
	Inner lesions	4.1 (3.2)	3.3 (2.3)	5.9 (4.0)	**<0.001^a^**
	Deep lesions	6.3 (7.2)	4.6 (4.5)	10.3 (10.0)	**<0.001^a^**
	Outer lesions	3.3 (3.3)	2.4 (2.1)	5.3 (4.4)	**<0.001^a^**
	Infratentorial	0.01 (0.04)	0.01 (0.04)	0.01 (0.03)	0.95 ^a^
*Brain and spinal cord volumes [mL]* *					
	Total brain volume	1136.4 (110.9)	1147.5 (110.5)	1111.4 (108.3)	**0.019^a^**
	Basal Ganglia	34.3 (3.5)	34.9 (3.3)	33.0 (3.7)	**<0.001^a^**
	Hippocampus	7.6 (0.7)	7.7 (0.7)	7.5 (0.8)	**0.02 ^a^**
	Thalamus	10.1 (1.5)	10.5 (1.3)	9.2 (1.5)	**<0.001^a^**
	Cingulate	27.6 (3.3)	27.8 (3.3)	27.3 (3.3)	0.256 ^a^
	Frontal lobe	180.4 (19.2)	182.9 (19.1)	174.8 (18.4)	**0.002 ^a^**
	Insula	10.7 (1.3)	10.9 (1.3)	10.2 (1.1)	**<0.001^a^**
	Occipital lobe	66.9 (8.3)	68.3 (8.1)	63.9 (8.0)	**<0.001^a^**
	Parietal lobe	91.3 (10.6)	92.5 (10.6)	88.7 (10.1)	**0.01 ^a^**
	Temporal lobe	128.2 (13.5)	129.5 (13.6)	125.0 (12.9)	**0.02 ^a^**
	Cerebellar grey matter	94.5 (9.5)	95.4 (9.5)	92.6 (9.1)	**0.031^a^**
	MUCCA	64.5 (7.9)	65.7 (7.6)	61.6 (8.0)	**0.001^a^**
*Functional hubness (EC [z-scores])*					
	Basal ganglia network	−0.020 (0.242)	−0.026 (0.252)	−0.006 (0.216)	0.549 ^a^
	Default mode network	0.007 (0.213)	−0.013 (0.223)	0.052 (0.184)	**0.027**^a^
	Sensorimotor cortex network	−0.042 (0.222)	−0.037 (0.227)	−0.053 (0.209)	0.600 ^a^
*White matter tract integrity (FA [0*–*1])*					
	Anterior thalamic radiation	0.45 (0.04)	0.46 (0.03)	0.43 (0.04)	**<0.001^a^**
	Corticospinal tract	0.65 (0.03)	0.66 (0.03)	0.64 (0.03)	**0.002 ^a^**
	Cingulum	0.56 (0.05)	0.58 (0.04)	0.54 (0.06)	**<0.001^a^**
	Other WM tracts	0.53 (0.04)	0.54 (0.03)	0.50 (0.05)	**<0.001^a^**

* Mean (standard deviation), ^**^ number (percentage), ^***^ median (IQR). ^a^ Independent-samples *t*-test, ^b^ chi-square test, ^c^ Mann-Whitney *U* test.

CI: cognitively impaired; CP: cognitively preserved; DMT: disease modifying treatment, EDSS: expanded disability status scale, IQR: interquartile range, MCI: mild cognitively impaired.

Table 3 shows the clinical measures of the patient group split into cognitively preserved and cognitively impaired.

We used SPSS 22.0 and 24.0 (IBM Corp., Armonk, NY, USA) and the scipy python package (version 1.2.1) for statistical analyses. The level of significance for demographic and clinical data (Table 1, Table 2, Table 3) was set at *p <* 0.05.

### 2.7 Model fitting

The dEBM relies on a Gaussian Mixture of the biomarker distributions, and requires a sufficient separation of the respective distributions from the control and disease groups. Therefore, we performed a biomarker post-selection and included only those biomarkers that passed a two-sided independent samples *t*-test at a significance level of *p* ≤ 0.1. We used 1000 bootstraps sampled from the same cohort in order to estimate the positional variance of the event sequence. Individual subjects were finally staged within the model between stage 0 (no abnormal biomarkers) and stage *N* (all *N* biomarkers are abnormal).

Three dEBMs were built to characterize the structural, functional, and cognitive changes in ROMS progression generally (Model 1), and specifically for disability worsening (Model 2) and cognitive decline (Model 3).

• Model 1: Event sequence in all ROMS patients as a progression from HC.
• Model 2: Event sequence in ROMS patients progressing from low (EDSS 0.0 – 2.5) to high disability level (EDSS ≥ 4.0). Intermediate patients with an EDSS of 3.0 or 3.5 were excluded from the GMM initialisation but used to estimate the event sequence. HCs were excluded for this analysis.
• Model 3: Event sequence in MS patients progressing from cognitively preserved (CP) to cognitively impaired (CI). Patients with mild cognitive impairment (MCI) were excluded from the GMM initialisation but used to estimate the event sequence. HCs were excluded for this analysis.

## 3 Results

**Fig. 1:**
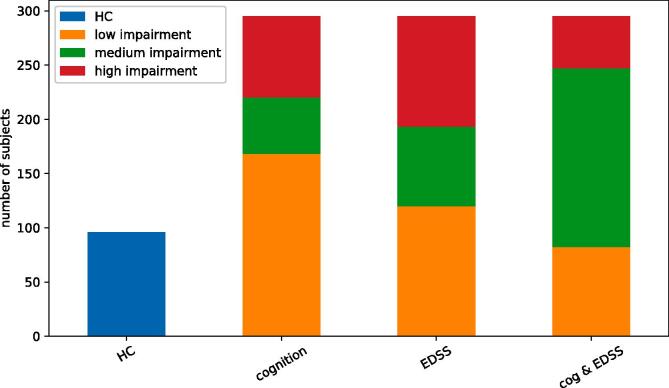
Overview of diagnostic groups, and separation of ROMS subgroups.

At the time of data acquisition, 243 of ROMS patients were diagnosed with relapsing-remitting MS (RRMS) and 52 patients with secondary progressive MS (SPMS). The average age was 46.7 (standard deviation 11.0) years and patients had their symptom onset 12.6 ± 1.6 years prior to assessment. The proportion of women was higher in the patient group (71.5%) than in HCs (58.3%, *p* = 0.016) and HCs had a higher educational level (*p* = 0.017). The median EDSS was 3.0 (IQR 2 – 4) with 120 patients having low disability (EDSS 0.0 – 2.5) and 102 patients having high disability (EDSS ≥ 4.0). Seventy-five patients were cognitively impaired (CI), 52 patients were classified as MCI and 168 patients as CP (Fig. 1). Demographics and MRI metrics of patients and HCs are shown in Table 1. For the MRI measures, only two functional networks (DMN and basal ganglia) were not significantly different between patients and healthy controls after correction for confounding variables.

Biomarker post-selection resulted in 21, 25, and 17 biomarkers included in the final models 1 (general MS progression), 2 (disability in MS) and 3 (cognitive decline in MS), respectively.

**Fig. 2:**
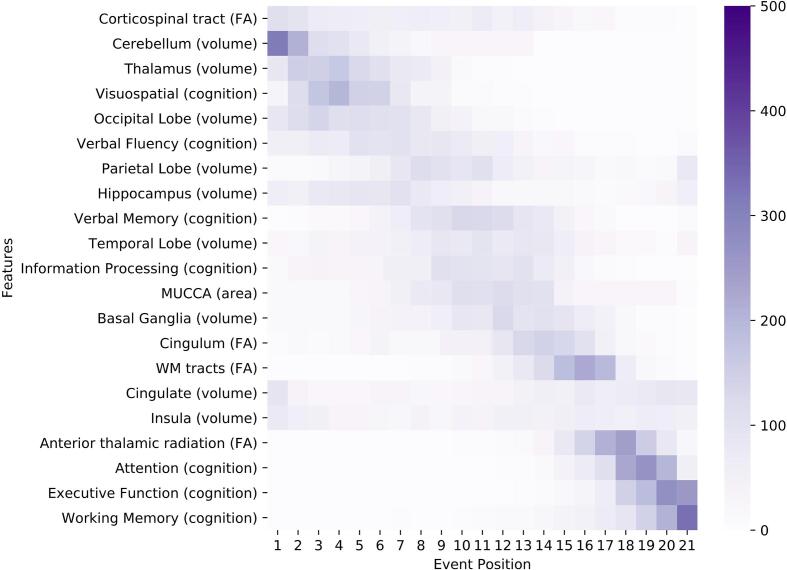
Positional variance diagram for the general ROMS population (Model 1). The maximum-likelihood sequence of abnormality is shown on the y-axis (top to bottom). Colour intensity in each row indicates positional variance: the darker the colour, the higher the confidence of the event position across 1000 bootstraps (capped at 500 for visualisation). The biomarker ordering reflects the sequence obtained from fitting all subjects. EC = eigenvector centrality; EDSS: expanded disability status scale; FA: fractional anisotropy as a measure for microstructural WM tract changes; MUCCA: mean upper cervical cord area.

We visualize the models using positional variance diagrams (PVD; see Fig. 2, Fig. 4, Fig. 6). The positional variance diagram shows the most likely sequence of events on the y-axis, while the x-axis represents the event position within the sequence ranging from one to the number of events. The intensity of each field represents the number of bootstraps where an event appeared at that respective position. This indicates uncertainty in the sequence, such that a strong confidence in the ordering results in a dark diagonal in the positional variance diagram.

**Fig. 3:**
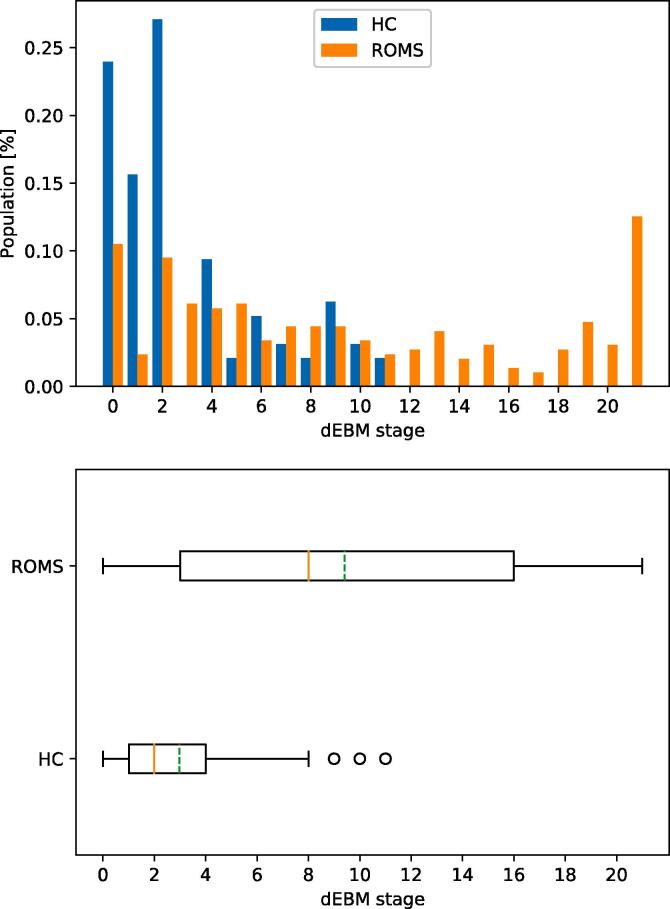
Patient staging for Model 1 (ROMS). Top: Staging of HC and ROMS subjects within the 21 disease stages. Bottom: Boxplot of staging indicating median (solid red line) and mean (dashed green line) of the groups. (For interpretation of the references to colour in this figure legend, the reader is referred to the web version of this article.)

**Fig. 4:**
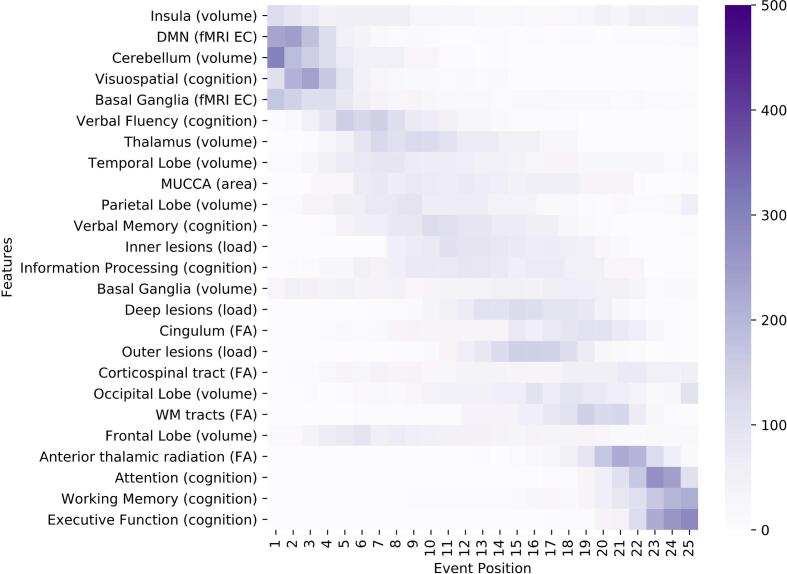
Positional variance diagram for the progression from low to high disability in ROMS patients (Model 2). The maximum-likelihood sequence of abnormality is shown on the y-axis (top to bottom). Colour intensity in each row indicates positional variance: the darker the colour, the higher the confidence of the event position across 1000 bootstraps (capped at 500 for visualisation). The biomarker ordering reflects the sequence obtained from fitting all subjects. EC = eigenvector centrality; EDSS: expanded disability status scale; FA: fractional anisotropy as a measure for microstructural WM tract changes; MUCCA: mean upper cervical cord area.

**Fig. 5:**
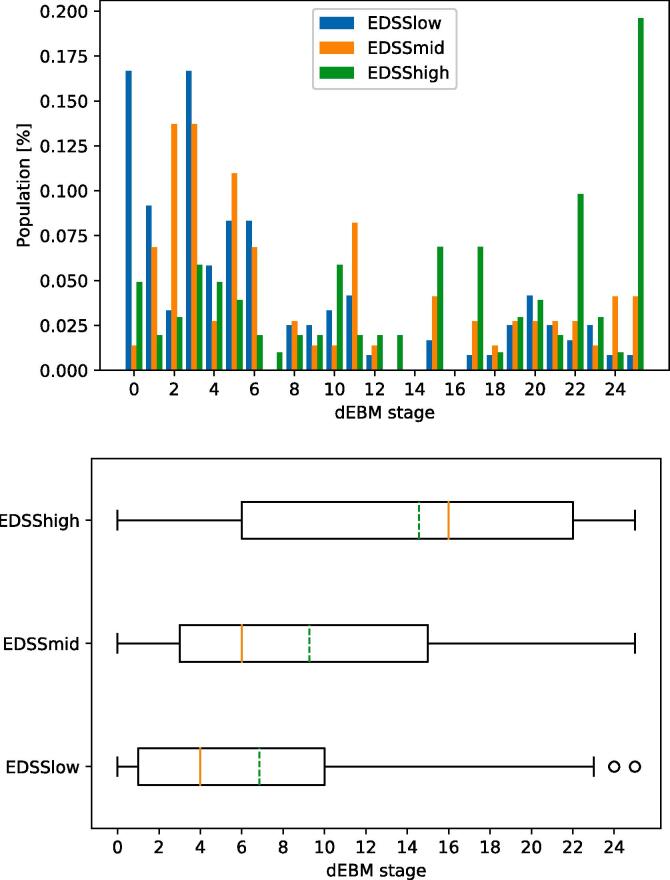
Patient staging for Model 2 (disability). Top: Staging of subjects with different levels of disability within the 25 disease stages. Bottom: Boxplot of staging indicating median (solid red line) and mean (dashed green line) of the groups. (For interpretation of the references to colour in this figure legend, the reader is referred to the web version of this article.)

**Fig. 6:**
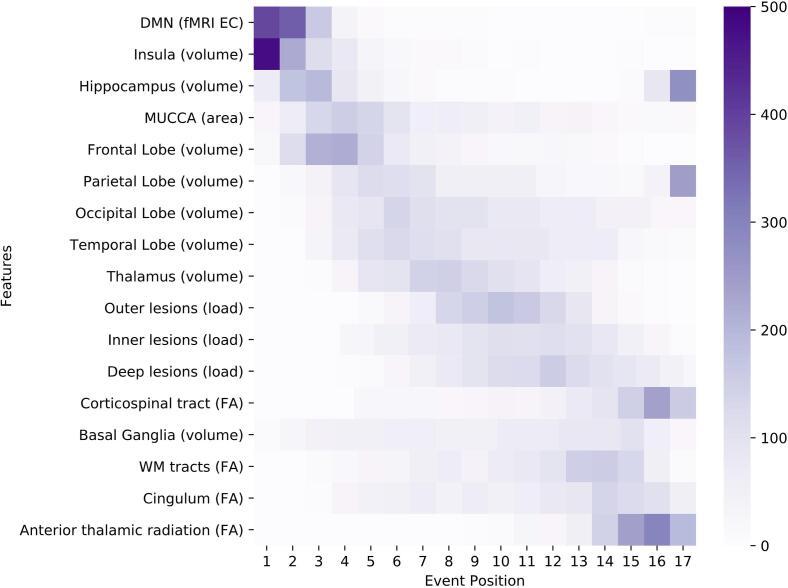
Positional variance diagram for the progression in ROMS patients as cognition declines (Model 3). The maximum-likelihood sequence of abnormality is shown on the y-axis (top to bottom). Colour intensity in each row indicates positional variance: the darker the colour, the higher the confidence of the event position across 1000 bootstraps (capped at 500 for visualisation). The biomarker ordering reflects the sequence obtained from fitting all subjects. EC = eigenvector centrality; EDSS: expanded disability status scale; FA: fractional anisotropy as a measure for microstructural WM tract changes; MUCCA: mean upper cervical cord area.

### 3.1 Model 1: Sequence of events in relapse-onset multiple sclerosis progression

The PVD of Model 1 is shown in Fig. 2. Despite considerable uncertainty, ROMS tends to starts with decreases in the corticospinal tract FA as well as cerebellar and thalamic atrophy. Neurodegeneration continues to involve the occipital and parietal lobes (position 5 and 7 of 21), through the temporal lobe, spinal cord (MUCCA) and basal ganglia (position 10, 12 and 13 of 21), with the cingulate and insula being affected later (position 16–17 of 21). Deficiency in visuospatial cognition is the earliest cognitive abnormality at position 4, shortly after thalamic atrophy, followed by verbal fluency, verbal memory and information processing (position 6, 9 and 11 of 21). Other cognitive domains are estimated to be affected later. FA changes of the cingulum and the non-specific WM-tracts appear in the last third of the event sequence between the basal ganglia and the cingulate volume events. Anterior thalamic radiation FA becomes abnormal late (position 18 of 21).

The staging reveals that healthy controls are mostly placed at earlier stages and no HC being staged higher than stage 11 of 21 (median stage 2, mean stage 3) while ROMS patients are spread across all stages with a median stage of 8 (mean 9.4) as shown in Fig. 3.

The effect on leaving out individual biomarkers or groups of biomarkers from a certain modality is very small as shown qualitatively in the Supplementary Materials.

The PVD for the main tracts of the JHU WM tractography atlas is shown in Figure S4.

### 3.2 Model 2: Sequence of events in the progression of low-to-high disability in ROMS

Table 2 shows the comparison between patients with high disability (EDSS of 4.0 or higher, n = 102) and patients with low disability (EDSS of 2.5 or lower, n = 120). Patients with high disability were older (average 53.1 versus 41.0 years, *p* < 0.001), had longer symptom duration (average 18.8 versus 10.6 years, *p* < 0.001), had a lower level of education (5 versus 4, *p* = 0.001), and a higher percentage of cognitive impairment (47.1% versus 13.3%, *p* < 0.001) than patients with low disability. Not all MRI measures showed significant differences (*p* < 0.1 was accepted in the biomarker post-selection) between patients with high versus low disability. The included markers are listed in Fig. 4.

The sequence for progression from low to high disability is shown in Fig. 4. Insular and cerebellar GM atrophy occur early in the event sequence together with changes in centrality of the default-mode and basal-ganglia networks, and visuospatial perception (position 1–5 of 25). Atrophy continues to occur in the thalamus, temporal lobe, MUCCA, parietal lobe, basal ganglia, while occipital and frontal lobe atrophy occur relatively late (position 19 and 21 of 25 respectively). Lesion load becomes abnormal first in the inner (periventricular) regions, then in the deep WM and the outer regions (i.e. juxtacortical). Changes in the FA biomarkers appear in the last third of the sequence, and cognitive changes in attention, working memory and executive function are last to become abnormal.

The patient staging shows that ROMS patients with all levels of disability can be found in all 25 stages (see Fig. 5). However, there is a clear trend such that patients with low EDSS have a median stage of 4 (mean 6.9), patients with medium disability have a median stage of 6 (mean 9.3), and patients with a high level of disability have a median stage of 16 (mean 14.6).

### 3.3 Model 3: Sequence of events in ROMS as cognition declines

All 295 patients had complete cognitive tests: 75 patients were classified as CI, 52 as MCI, and 168 patients as CP. Patients with CI were older (average 50.4 versus 45.7 years; *p* = 0.001), had a longer symptom duration (average 17.6 versus 13.3 years, *p* < 0.001), had a lower educational level (4 versus 6, *p* < 0.001) and a higher EDSS score (median 4.0 versus 3.0; *p* < 0.001), see Table 3 for the comparisons between CI and CP patients.

The ordering of events in the dEBM of cognitive impairment is shown in Fig. 6. Similar to Model 2, the progression in cognitive decline is accompanied by early insular atrophy and increased functional DMN centrality. The event sequence continues with atrophy of the hippocampus, cervical cord, frontal, parietal, occipital and temporal lobes, and the thalamus (position 3–9 of 17) and finally the basal ganglia (position 14 of 17). Lesion events occur in close succession after most atrophy measures (position 10–12 of 17). Changes in WM tract FA occur at the end with the corticospinal tract being affected earlier than the rest (position 13 of 17).

**Fig. 7:**
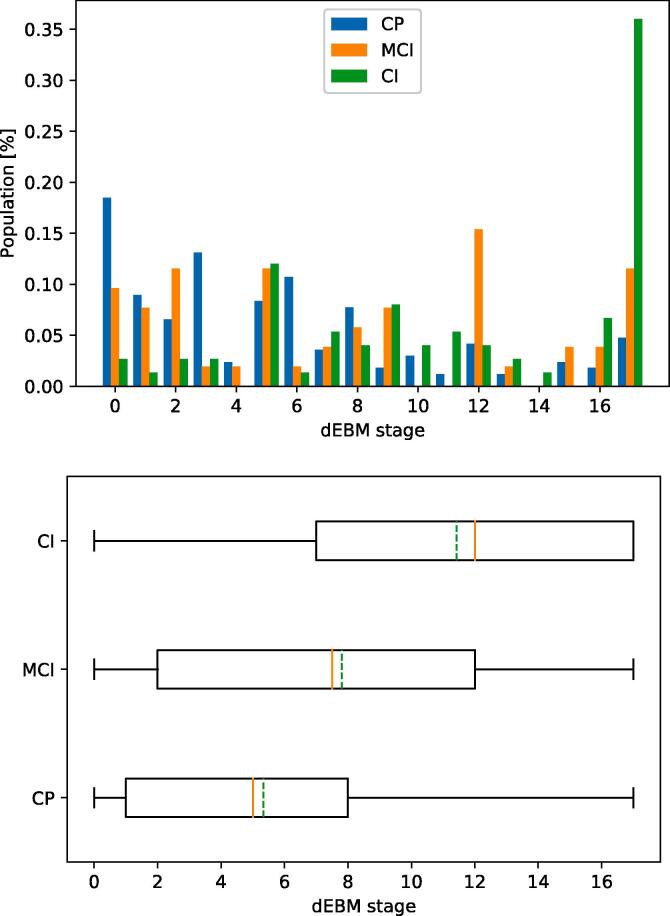
Patient staging for Model 3 (cognition). Top: Staging of subjects with different levels of cognitive abilities within the 17 disease stages. Bottom: Boxplot of staging indicating median (solid red line) and mean (dashed green line) of the groups. (For interpretation of the references to colour in this figure legend, the reader is referred to the web version of this article.)

As in Model 2, all three groups are spread across all stages (see Fig. 7). Cognitively preserved ROMS patients have a median stage of 5 (mean 5.3), patients with MCI have a median stage of 7.5 (mean 7.8), and cognitively impaired patients have a median stage of 12 (mean 11.4).

## 4 Discussion

Current understanding of disease progression in MS is largely based on studies that each considered a small number of MS pathology features in isolation. This body of work has identified lesion number and location, regional atrophy, changes in functional centrality of brain networks, or alterations in WM tract microstructure as features of interest. Until now, the sequence of accumulated abnormality in these biomarkers relative to each other remained largely undetermined. Our data-driven dEBM analysis suggests that changes of the corticospinal tract, and GM volume changes of cerebellum, thalamus and occipital lobe are early events; whereas microstructural changes in other WM tracts and changes in cognitive domains attention, executive function and working memory are relatively late events in MS progression. We also estimated sequences specific to disability worsening and cognitive impairment motivated to reveal new insight into the underlying mechanisms of each, and to provide a quantitative template for patient assessment. The results of this secondary analysis suggest that functional network centrality of the default mode network is involved early in both, with DTI-related WM tract abnormality occurring later.

### 4.1 Model 1: Sequence of events in relapse-onset multiple sclerosis progression

The general ROMS model suggests that cerebellar atrophy is an early event. Although studies in patients with a clinically isolated syndrome (CIS) have not been conclusive on the presence of early cerebellar volume loss (Parmar, 2018), a recent EBM study in MS patients also showed cerebellar atrophy as part of the atrophy sequence (Eshaghi et al., 2018). Early thalamic and hippocampal atrophy in our study are in accordance with findings in previous studies reporting atrophy in these areas already in CIS patients (Audoin, 2010, Henry, 2008). Insular and cingulate atrophy occur relatively late in our study but the bootstrap analysis shows a bimodal distribution for these biomarkers with clusters at the beginning and the end of the sequence (see Fig. 2), which might indicate heterogeneity in the population such that some patients have the event early whereas others experience this later. Among the volumetric measurements, MUCCA abnormality occurs at an intermediate position in the event ordering, while previous literature indicates that spinal cord atrophy can be seen already in CIS patients on a group level and with high clinical relevance (Biberacher, 2015). This could be due to differences in measurement sensitivity or cohort size and requires further study.

A second marker of white matter abnormality, the FA of the corticospinal tract, appears as the first event, which agrees with previous work (Biberacher, 2015). In addition, research found that WM changes occur early in the disease (Huang et al., 2018), whereas most FA markers other than corticospinal tract damage included in this study were late events in the model. A probable explanation could be that the WM damage from lesions within the tracts is relatively small compared to overall sizes of the tract ROIs, so that the tract features mainly represent normal-appearing WM and hence have little disease signal. Additionally, there is substantial inter-patient heterogeneity in the anatomical distribution of WM damage, which creates an unclear relation between microstructural changes in specific WM tracts and progression along the disease course.

The seven included cognitive domains are spread across the progression timeline but previous literature does not provide many concrete indications regarding the true positioning of those biomarkers given the lack of longitudinal data. However, the domains attention, executive function, and working memory were consistently late events in our analyses, which is supported by previous research (Meijer, 2016). Overall, we showed that the obtained event sequence is well in line with previous work on individual features but provides additional insight in the relative positioning of the multimodal features. The obtained sequence can potentially be used to stage patients within the disease course and help with clinical monitoring of disease progression beyond relapses and physical disability. However, the relatively high uncertainty limits use for individual patients at this stage.

### 4.2 Model 2: Sequence of events in the progression of low-to-high disability in relapse-onset multiple sclerosis

The model for progression from low disability to high disability has many similarities to the general MS event sequence (Model 1) such as the early occurrence of cerebellar atrophy or visuospatial memory impairment, and the late events for white matter tract FA and the cognitive domains of attention, working memory and executive function. This is somewhat expected as minor impairment starts early in the disease course when brain structure is most similar to healthy controls.

The most notable difference with Model 1 is the early increase of eigenvector centrality of the DMN and basal ganglia functional network, which supports findings on functional centrality as a correlate of physical disability (Schoonheim et al., 2014). Similarly, basal ganglia atrophy appears early in Model 2, supporting recent findings of deep GM atrophy being a driving factor in disability worsening (Eshaghi et al., 2018). The insula appears to be the earliest event but the considerable uncertainty suggests variability between individuals.

Changes of the MUCCA measurement appear earlier and FA changes of the corticospinal tract appear later with respect to Model 1. This ostensibly contradictory finding could be interpreted such that initial damage of the corticospinal tract already occurred in patients with low disability (i.e. first event in the progression from HC to MS) and more severe damage (i.e. spinal cord atrophy) will become apparent later. At the same time the cord area is not strongly affected initially but changes become more detectable after MS onset has occurred as indicated by previous studies that have shown the relevance of spinal cord atrophy in explaining long-term disability (Daams, 2014, Lukas, 2015).

The thalamus is broadly involved in cognitive and sensorimotor functions (Tewarie, 2015), which could explain the very early position in Model 1 and an early position in Model 2, and can be interpreted as a further increase in abnormality alongside the increase in disability.

MS lesions appear to become significant towards the cortex as disability progresses, i.e. first in the periventricular white matter, then in the deep WM and finally closer to the cortex, which is in line with other studies showing a larger lesion load around the ventricles with fewer lesions juxtacortically (Rossi et al., 2012, Giorgio et al., 2013). It should be noted, however, that this study does not include measurements of cortical lesions, which needs to be addressed in subsequent studies.

### 4.3 Model 3: Sequence of events in relapsing-onset multiple sclerosis as cognition declines

In the dEBM sequence from CP to CI, early events were atrophy of the insula, hippocampus and spinal cord, as well as the increased functional centrality of the DMN. The early appearance of insular atrophy in this model is interesting in the light of previous studies showing the fastest volume loss in these areas in patients with SPMS (Eshaghi et al., 2018, Liu, 2014). We infer that these volume changes are an early event in the general MS population, confirmed by their respective positioning in a previous EBM study sequence (Eshaghi et al., 2018).

A meaningful comparison of the functional centrality of networks is impeded by the exclusion of the basal ganglia and sensorimotor network biomarkers from the model due to statistically indistinguishable biomarker distributions between CP and CI groups, indicating that these have limited relevance to cognitive decline in MS. However, the increased functional centrality of the DMN was an early event in both model 2 and 3 suggesting that abnormality of DMN functional centrality could be an early indication of future cognitive and physical decline, as has been suggested extensively in MS literature (Eijlers et al., 2017, Schoonheim et al., 2015).

The interpretation and relevance of the early positioning of MUCCA in the cognitive model is difficult to understand but might reflect the overlap between patients with CI and patients with increased physical disability (64% of patients with CI in this cohort also have more severe physical disability; see also Table 2, Table 3 and Fig. 1). Lesion events appear in direct succession and the positional variance diagram (Fig. 6) indicates that abnormal lesion volumes occur in all three locations roughly at the same time, indicating that other measures such as atrophy and brain function are more important for cognition.

Though thalamic atrophy has been associated with cognitive decline and disease progression (Schoonheim et al., 2012), it appears relatively later (mid-sequence) than expected in the dEBM sequence. This could be the result of a floor-effect as there is already thalamic atrophy present in CP patients (Henry, 2008) and further changes arise late in the progression from CP to CI. Microstructural WM changes appear late in model 3, which is consistent with model 2 and could imply that these measures reflect advanced stages of disease progression. A previous study showed that only CI patients with atrophy had microstructural WM changes and CP patients without atrophy did not have WM tract abnormalities (Eijlers et al., 2018). Alternatively, the order in which different tracts become abnormal varies and more tracts are affected with advanced disease (Huang et al., 2018).

### 4.4 Considerations regarding features in the models

White matter lesions are a sensitive indicator for MS diagnosis (Thompson, 2018) and are used extensively in daily clinical practice. We analysed lesion locations at three depths, with the inner band including the lesions close to the ventricles, the outer band including those close to the cortex, and the intermediate deep WM lesions in between (Pardini et al., 2016). While this definition is not as stringent as the clinically used stratification into periventricular, juxtacortical and deep lesions, it is a useful approximation that can be derived in a consistent and data-driven fashion. Infratentorial lesions were only present in a small subset of patients and were therefore discarded from further analysis despite their involvement in clinical disability. Although minor (vascular) WM lesions could be present in controls, these lesions could not be included due to the lack of FLAIR imaging in controls. As such, in the analysis of general ROMS progression, Model 1, we did not include lesions. However, lesions would be expected to occur very early in the MS sequence. MUCCA measurement was performed using SCT-PropSeg on 3DT1 head images, which may have reduced sensitivity to change compared to dedicated cervical cord imaging although several studies have shown good agreement between MUCCA derived from head and cervical images (Lukas et al., 2018, Liu, 2016). We note that the considerable positional variance in the estimated ordering means that the exact positions of events should be interpreted with caution. Additionally, the ordering does not imply causation.

While we took care to include biomarkers of relevance to MS pathology, many more candidate biomarkers could be included in the future. Features such as spinal cord lesions (Sombekke et al., 2013), cerebrospinal fluid alterations (Disanto, 2017), or (semi)quantitative MR measures of myelination such as magnetization transfer ratio (MTR) have been shown to be sensitive to the MS pathogenesis but were unavailable in this cohort.

### 4.5 Study limitations

MS is a heterogeneous disease with multiple concurrent disease processes, which are difficult to model, especially with limited data. As a consequence, some biomarkers show clear bimodal behaviour in the positional variance diagram (e.g., cingulate and insular atrophy in Model 1), which suggests different orderings for subgroups of our cohort. While this impedes interpretation of some results, we believe that it is an important finding. An alternative way to model heterogeneous trajectories is to use advanced data-driven subtyping models such as SuStaIn (Young, 2018), which could potentially identify clusters of subjects that share a differential sequence of events and hence model the disease progression in MS more reliably. However, this typically requires a larger dataset than is available here.

The effects of disease modifying treatment is very challenging to model due to the heterogeneity in the disease progression and the resulting treatment options. In general, we would expect a reduction of EDSS or lesion occurrence as these are the main outcome measures for clinical trials. In this study, this would lead to a change in group assignments, especially for Model 2, but we would not expect a strong effect on other biomarkers or their event sequence. A comparison of sequences obtained from treated and untreated patients, as well as the effect of a complex statistical correction for treatment effects, should be performed in an independent and sufficiently large cohort.

EBM provides a temporal ordering of biomarker abnormality, but no actual information about time as the intervals between subsequent events are not linear; this means that the division into late and early events can only be interpreted relative to other markers within the overall disease course. A combination of EBM-type models with longitudinal data and survival models, however, could give an estimate of the timescales of disease progression (Young et al., 2014, Venkatraghavan et al., 2019).

## 5 Conclusion

This study has revealed the sequence of observable (biomarker) changes in brain structure, function, and cognition in the progression of ROMS, including specific sequences associated with disability worsening and cognitive decline. In general, changes in GM volume, especially of the thalamus, insula, hippocampus and cerebellum were the earliest events in MS and MS-related physical disability and cognitive decline, which also showed strong involvement of default-mode dysfunction. Microstructural changes in WM tracts were predominantly late events, which deserves further investigation as it appears to contradict the early occurrence of focal white matter lesions in many tracts, possibly indicating that overall tract integrity is maintained for a longer period of time. The relatively high uncertainty could be reduced using advanced models taking into account multiple concurrent disease trajectories within one cohort. Future research should also include patients soon after first symptoms arise (i.e., CIS) to determine the earliest disease pathologies in MS with high certainty.

## 6 Study funding

This project has received funding from the European Union’s Horizon 2020 research and innovation programme under grant agreement No. 666992, and was sponsored by the Dutch MS Research Foundation, grant numbers 08-650, 13-820 and 14-358e. FB, DCA, and NPO are supported by the NIHR Biomedical Research Centre at UCLH.

## 7 Statistical analysis

Statistical analysis was performed by I. Dekker, MD, Neil. P. Oxtoby, PhD, Daniel C. Alexander, PhD, Viktor Wottschel, PhD.

## CRediT authorship contribution statement

**Iris Dekker:** Conceptualization, Formal analysis, Writing - original draft, Writing - review & editing. **Menno M. Schoonheim:** Data curation, Conceptualization, Writing - review & editing. **Vikram Venkatraghavan:** Conceptualization, Formal analysis, Software, Writing - review & editing. **Anand J.C. Eijlers:** Data curation, Writing - review & editing. **Iman Brouwer:** Formal analysis, Methodology, Writing - review & editing. **Esther E. Bron:** Writing - review & editing. **Stefan Klein:** Writing - review & editing. **Mike P. Wattjes:** Data curation, Writing - review & editing. **Alle Meije Wink:** Data curation, Writing - review & editing. **Jeroen J.G. Geurts:** Data curation, Writing - review & editing. **Bernard M.J. Uitdehaag:** Data curation, Writing - review & editing. **Neil P. Oxtoby:** Conceptualization, Funding acquisition, Project administration, Writing - review & editing. **Daniel C. Alexander:** Conceptualization, Funding acquisition, Project administration, Writing - review & editing. **Hugo Vrenken:** Conceptualization, Writing - review & editing. **Joep Killestein:** Data curation, Writing - review & editing. **Frederik Barkhof:** Conceptualization, Data curation, Funding acquisition, Supervision, Writing - review & editing. **Viktor Wottschel:** Conceptualization, Data curation, Formal analysis, Methodology, Project administration, Supervision, Validation, Visualization, Writing - original draft, Writing - review & editing.

## Conflicts of interest

**Dekker** received speaking honoraria from Roche. **M.M. Schoonheim** serves on the editorial board of Frontiers of Neurology and has received compensation for consulting services or speaker honoraria from ExceMed, Genzyme and Biogen. **V. Venkatraghavan** reports no disclosures. **A.J.C. Eijlers** reports no disclosures. **I. Brouwer** reports no disclosures. **E.E. Bron** reports no disclosures. **S. Klein** reports no disclosures. **M.P. Wattjes** reports personal consulting/speaking fees from Biogen, Novartis, Roche, Celgene, IXICO, Sanofi Genzyme, Bayer Healthcare, Biologix, Genilac, Merck Serono. **A.M. Wink** reports no disclosures. **J.J.G. Geurts** is an editor of MS journal and serves on the editorial boards of Neurology and Frontiers of Neurology and is president of the Netherlands organization for health research and innovation and has served as a consultant for Merck-Serono, Biogen, Novartis, Genzyme and Teva Pharmaceuticals. **B.M.J. Uitdehaag** reports personal fees from Genzyme, Biogen Idec, TEVA, Merck Serono, Roche, outside the submitted work. **N.P. Oxtoby** reports no disclosures. **D.C. Alexander** reports no disclosures. **H. Vrenken** has received research grants from MerckSerono, Teva and Novartis, consulting fees from MerckSerono, and speaker honoraria from Novartis; all paid directly to his institution. **J. Killestein** reports grants and personal fees from Biogen Idec, Novartis, Merck Serono, TEVA, Genzyme, grants and other from Biogen Idec, Novartis, TEVA, Bayer Schering Pharma, Glaxo Smith Kline, Merck Serono. **F. Barkhof** reports grants and personal fees from Roche, Biogen Idec, Novartis, Merck Serono, TEVA, IXICO, grants and other support from Biogen Idec, Novartis, GE healthcare and Merck Serono. **V. Wottschel** reports no disclosures.

## References

[b0005] Amato M.P. (2006). The Rao’s Brief Repeatable Battery and Stroop Test: normative values with age, education and gender corrections in an Italian population. Mult. Scler..

[b0010] Audoin B. (2010). Atrophy mainly affects the limbic system and the deep grey matter at the first stage of multiple sclerosis. J. Neurol. Neurosurg. Psychiatry.

[b0015] Bergsland N. (2012). Subcortical and cortical gray matter atrophy in a large sample of patients with clinically isolated syndrome and early relapsing-remitting multiple sclerosis. AJNR. Am. J. Neuroradiol..

[b0020] Biberacher V. (2015). Atrophy and structural variability of the upper cervical cord in early multiple sclerosis. Mult. Scler..

[b0025] Cardoso M.J., Modat M., Wolz R., Melbourne A., Cash D., Rueckert D., Ourselin S. (2015). Geodesic information flows: spatially-variant graphs and their application to segmentation and fusion. IEEE Trans. Med. Imaging.

[b0030] Compston A., Coles A. (2008). Multiple sclerosis. The Lancet.

[b0035] Daams M. (2014). Mean upper cervical cord area (MUCCA) measurement in long-standing multiple sclerosis: relation to brain findings and clinical disability. Mult. Scler..

[b0040] De Leener B., Kadoury S., Cohen-Adad J. (2014). Robust, accurate and fast automatic segmentation of the spinal cord. NeuroImage.

[b0045] Dekker I., Wattjes M.P. (2017). Brain and Spinal Cord MR Imaging Features in Multiple Sclerosis and Variants. Neuroimaging Clin. N. Am..

[b0050] Disanto G. (2017). Serum Neurofilament light: A biomarker of neuronal damage in multiple sclerosis. Ann. Neurol..

[b0055] Eijlers A.J.C. (2018). Predicting cognitive decline in multiple sclerosis: A 5-year follow-up study. Brain.

[b0060] Eijlers A.J.C., Meijer K.A., Wassenaar T.M., Steenwijk M.D., Uitdehaag B.M.J., Barkhof F., Wink A.M., Geurts J.J.G., Schoonheim M.M. (2017). Increased default-mode network centrality in cognitively impaired multiple sclerosis patients. Neurology.

[b0065] Eijlers A.J.C., Meijer K.A., van Geest Q., Geurts J.J.G., Schoonheim M.M. (2018). Determinants of Cognitive Impairment in Patients with Multiple Sclerosis with and without Atrophy. Radiology.

[b0070] Eshaghi A. (2018). Deep gray matter volume loss drives disability worsening in multiple sclerosis. Ann. Neurol..

[b0075] Eshaghi, A., et al., 2018. Progression of regional grey matter atrophy in multiple sclerosis. Brain 141, 1665–1677. 10.1093/brain/awy088 PMC5995197 29741648

[b0080] Filippi, M., Charil, A., Rovaris, M., Absinta, M. Rocca, M.A., 2014. Insights from magnetic resonance imaging. in Handbook of Clinical Neurology, Elsevier B.V., 122, 115–149. 10.1016/B978-0-444-52001-2.00006-6 24507516

[b0085] Finke C. (2015). Altered basal ganglia functional connectivity in multiple sclerosis patients with fatigue. Mult. Scler..

[b0090] Fonteijn H.M., Modat M., Clarkson M.J., Barnes J., Lehmann M., Hobbs N.Z., Scahill R.I., Tabrizi S.J., Ourselin S., Fox N.C., Alexander D.C. (2012). An event-based model for disease progression and its application in familial Alzheimer's disease and Huntington's disease. NeuroImage.

[b0095] Giorgio A., Battaglini M., Rocca M.A., De Leucio A., Absinta M., van Schijndel R., Rovira A., Tintore M., Chard D., Ciccarelli O., Enzinger C., Gasperini C., Frederiksen J., Filippi M., Barkhof F., De Stefano N. (2013). Location of brain lesions predicts conversion of clinically isolated syndromes to multiple sclerosis. Neurology.

[b0100] Henry R.G. (2008). Regional grey matter atrophy in clinically isolated syndromes at presentation. J. Neurol. Neurosurg. Psychiatry.

[b0105] Huang J. (2018). White matter microstructural alterations in clinically isolated syndrome and multiple sclerosis. J. Clin. Neurosci. Off. J. Neurosurg. Soc. Australas..

[b0110] Ingala S. (2020). The relation between APOE genotype and cerebral microbleeds in cognitively unimpaired middle- and old-aged individuals. Neurobiol. Aging.

[b0115] Klein A., Tourville J. (2012). 101 Labeled Brain Images and a Consistent Human Cortical Labeling Protocol. Front. Neurosci..

[b0120] Koenig K.A. (2015). The relationship between cognitive function and high-resolution diffusion tensor MRI of the cingulum bundle in multiple sclerosis. Mult. Scler..

[b0125] Kurtzke, J.F., 1983. Rating neurologic impairment in multiple sclerosis: An expanded disability status scale (EDSS). Neurology 33, 1444–1444. 10.1212/wnl.33.11.1444 6685237

[b0130] Liu Y. (2014). Cortical Thinning Correlates with Cognitive Change in Multiple Sclerosis but not in Neuromyelitis Optica. Eur. Radiol..

[b0135] Liu Y. (2016). Multicenter Validation of Mean Upper Cervical Cord Area Measurements from Head 3D T1-Weighted MR Imaging in Patients with Multiple Sclerosis. AJNR. Am. J. Neuroradiol..

[b0140] Longo D.L., Reich D.S., Lucchinetti C.F., Calabresi P.A. (2018). Multiple sclerosis. N. Engl. J. Med..

[b0145] Lukas C. (2015). Cervical spinal cord volume loss is related to clinical disability progression in multiple sclerosis. J. Neurol. Neurosurg. Psychiatry.

[b0150] Lukas C. (2018). Quantification of spinal cord atrophy in MS: which software, which vertebral level, spinal cord or brain MRI? A multi-centric, longitudinal comparison of three different volumetric approaches. Mult. Scler. J..

[b0155] Meijer K.A. (2016). White matter tract abnormalities are associated with cognitive dysfunction in secondary progressive multiple sclerosis. Mult. Scler..

[b0160] Meijer K.A. (2018). Is impaired information processing speed a matter of structural or functional damage in MS?. NeuroImage. Clin..

[b0165] Mezzapesa D.M., Rocca M.A., Rodegher M., Comi G., Filippi M. (2008). Functional cortical changes of the sensorimotor network are associated with clinical recovery in multiple sclerosis. Hum. Brain Mapp..

[b0170] Oxtoby, N.P., et al., 2018. Data-driven models of dominantly-inherited Alzheimer’s disease progression. Brain 141, 1529–1544. 10.1093/brain/awy050 PMC5920320 29579160

[bib296] Mori (2005). MRI Atlas of Human White Matter.

[b0175] Pardini M. (2016). Relationship of grey and white matter abnormalities with distance from the surface of the brain in multiple sclerosis. J. Neurol. Neurosurg. Psychiatry.

[b0180] Parmar K. (2018). The role of the cerebellum in multiple sclerosis-150 years after Charcot. Neurosci. Biobehav. Rev..

[b0185] Polman C.H. (2011). Diagnostic criteria for multiple sclerosis: 2010 revisions to the McDonald criteria. Ann. Neurol..

[b0190] Prados F., Cardoso M.J., Kanber B., Ciccarelli O., Kapoor R., Gandini Wheeler-Kingshott C.A.M., Ourselin S. (2016). A multi-time-point modality-agnostic patch-based method for lesion filling in multiple sclerosis. NeuroImage.

[b0195] Rao, S.M., 1990. A manual for the brief repeatable battery of neuropsychological tests in multiple sclerosis. Medical College of Wisconsin.

[b0200] Reich D.S., Smith S.A., Zackowski K.M., Gordon-Lipkin E.M., Jones C.K., Farrell J.A.D., Mori S., van Zijl P.C.M., Calabresi P.A. (2007). Multiparametric magnetic resonance imaging analysis of the corticospinal tract in multiple sclerosis☆. NeuroImage.

[b0205] Rossi, F. et al. Relevance of brain lesion location to cognition in relapsing multiple sclerosis. PLoS One 7, e44826 (2012). 10.1371/journal.pone.0044826 PMC3489883 23144775

[b0210] Schoonheim M.M. (2014). Changes in functional network centrality underlie cognitive dysfunction and physical disability in multiple sclerosis. Mult. Scler..

[b0215] Schoonheim M.M., Popescu V., Rueda Lopes F.C., Wiebenga O.T., Vrenken H., Douw L., Polman C.H., Geurts J.J.G., Barkhof F. (2012). Subcortical atrophy and cognition: Sex effects in multiple sclerosis. Neurology.

[b0220] Schoonheim M.M., Hulst H.E., Brandt R.B., Strik M., Wink A.M., Uitdehaag B.M.J., Barkhof F., Geurts J.J.G. (2015). Thalamus structure and function determine severity of cognitive impairment in multiple sclerosis. Neurology.

[b0225] Schoonheim M.M., Meijer K.A., Geurts J.J.G. (2015). Network collapse and cognitive impairment in multiple sclerosis. Front. Neurol..

[b0230] Smith S.M., Jenkinson M., Woolrich M.W., Beckmann C.F., Behrens T.E.J., Johansen-Berg H., Bannister P.R., De Luca M., Drobnjak I., Flitney D.E., Niazy R.K., Saunders J., Vickers J., Zhang Y., De Stefano N., Brady J.M., Matthews P.M. (2004). Advances in functional and structural MR image analysis and implementation as FSL. NeuroImage.

[b0235] Sombekke M.H., Wattjes M.P., Balk L.J., Nielsen J.M., Vrenken H., Uitdehaag B.M.J., Polman C.H., Barkhof F. (2013). Spinal cord lesions in patients with clinically isolated syndrome: A powerful tool in diagnosis and prognosis. Neurology.

[b0240] Steenwijk M.D. (2013). Accurate white matter lesion segmentation by k nearest neighbor classification with tissue type priors (kNN-TTPs). NeuroImage Clin..

[b0245] Sudre C.H. (2018). Bullseye’s representation of cerebral white matter hyperintensities. J. Neuroradiol..

[b0250] Sudre C.H., Cardoso M.J., Ourselin S. (2017). Longitudinal segmentation of age-related white matter hyperintensities. Med. Image Anal..

[b0255] Tewarie P. (2015). Functional brain networks: linking thalamic atrophy to clinical disability in multiple sclerosis, a multimodal fMRI and MEG study. Hum. Brain Mapp..

[b0260] Thompson A.J. (2018). Diagnosis of multiple sclerosis: 2017 revisions of the McDonald criteria. Lancet Neurol..

[b0265] Venkatraghavan V., Bron E.E., Niessen W.J., Klein S. (2019). Disease progression timeline estimation for Alzheimer's disease using discriminative event based modeling. NeuroImage.

[b0270] Venkatraghavan, V., Bron, E., Niessen, W. Klein, S., 2017. A Discriminative Event Based Model for Alzheimer’s Disease Progression Modelingdssssssss. 10.1016/j.neuroimage.2018.11.024 30471388

[b0275] Verhage F., Van Der Werff J.J. (1964). An analysis of variance based on the groninger intelligence test scores. Ned. Tijdschr. Psychol..

[b0280] Wijeratne P.A. (2018). An image-based model of brain volume biomarker changes in Huntington’s disease. Ann. Clin. Transl. Neurol..

[b0285] Wink A.M., de Munck J.C., van der Werf Y.D., van den Heuvel O.A., Barkhof F. (2012). Fast Eigenvector Centrality Mapping of Voxel-Wise Connectivity in Functional Magnetic Resonance Imaging: Implementation, Validation, and Interpretation. Brain Connect..

[b0290] Young A.L. (2018). Uncovering the heterogeneity and temporal complexity of neurodegenerative diseases with Subtype and Stage Inference. Nat. Commun..

[b0295] Young, A.L., et al., 2014. A data-driven model of biomarker changes in sporadic Alzheimer’s disease. Brain 137, 2564–2577. 10.1093/brain/awu176 PMC4132648 25012224

