# Supplementary material for: The sequence of structural, functional and cognitive changes in multiple sclerosis
Source: Neuroimage Clin. 2020 Dec 24;29:102550. doi: 10.1016/j.nicl.2020.102550 (PMC7804841; doi:10.1016/j.nicl.2020.102550)
Supplement: Supplementary data 1 [file mmc1.docx]

**The sequence of structural, functional and cognitive changes in multiple sclerosis**

**Supplementary Material**

The combination of features from different domains, such as volumetric, FA or cognition, has no effect on the general event ordering, except for small variations within the uncertainty estimated by the bootstrap experiments. In the supplementary materials we present additional data from experiments with subsets of the biomarkers used in Model 1 to support this statement.

Exclusion of cognitive biomarkers

The event sequence obtained when using no cognitive biomarkers is almost identical (Figure S1) to the original ordering of Model 1 as shown in Figure 2, except for the excluded biomarkers, of course. The only difference is in the last three biomarkers Cingulate, Insula and Anterior thalamic radiation, where Cingulate and Anterior thalamic radiation have swapped position. This can be explained by the very high positional uncertainty of these biomarkers.


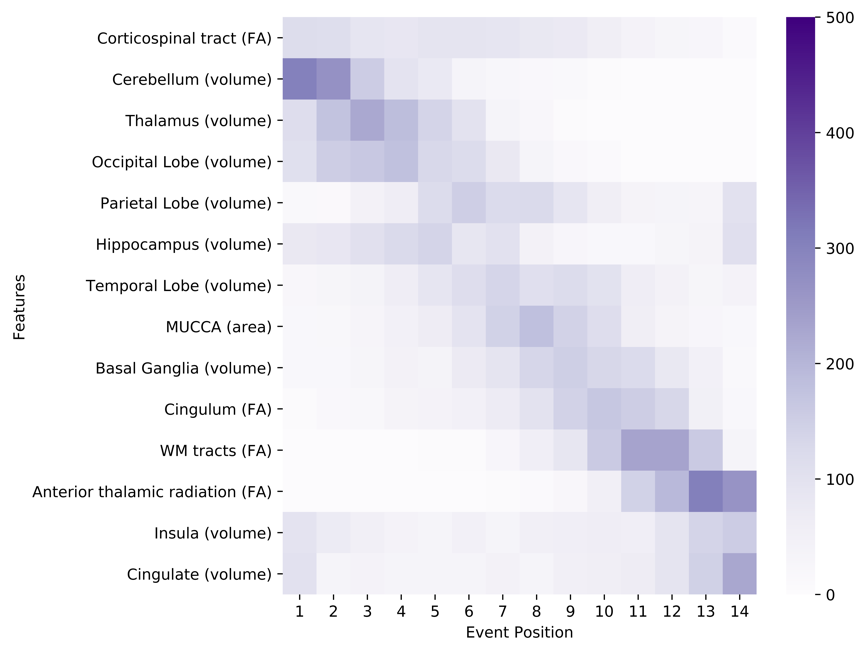


Figure S1: Positional variance diagram for subjects and biomarkers from Model 1 but excluding cognitive biomarkers. Please note that the colourbar is capped at 500 to improve visualisation.

Similarly, when creating an EBM using only cognitive biomarkers (Figure S2) we obtain an ordering that is alike to the ordering of cognitive features within the more comprehensive Model 1. Only ‘Verbal Memory’ and ‘Information Processing’, and ‘Executive Function’ and ‘Working Memory’ have swapped positions. These pairs of biomarkers are very close in event position, so this change is within the uncertainty estimated from the bootstraps.

**
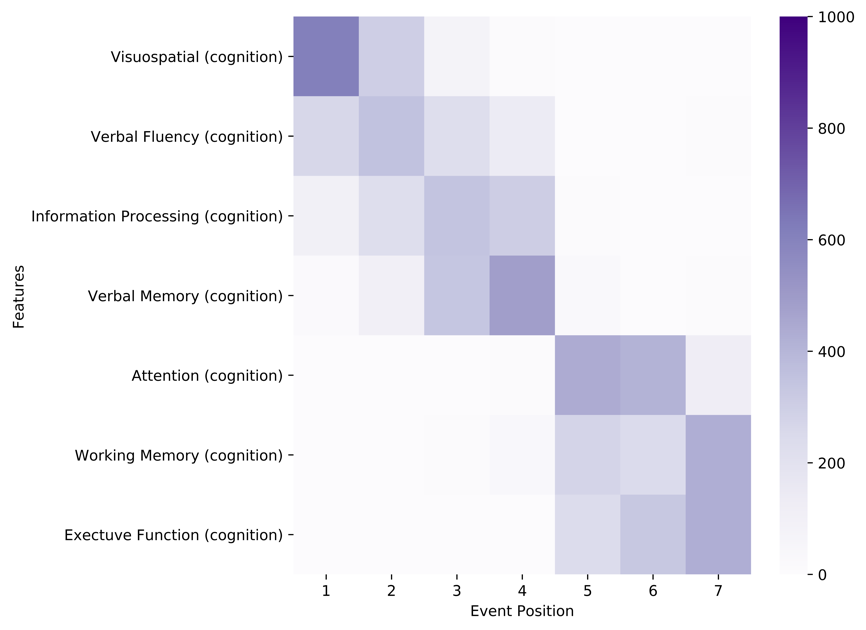
**

Figure S2: Positional variance diagram for subjects from Model 1 using only cognitive biomarkers.

Variation of included tracts for FA biomarkers

The relative ordering of the four tract-related biomarkers, when modelled independently of all other biomarkers as shown in Figure S3, is identical to the one obtained in Model 1 as shown in Figure 2.


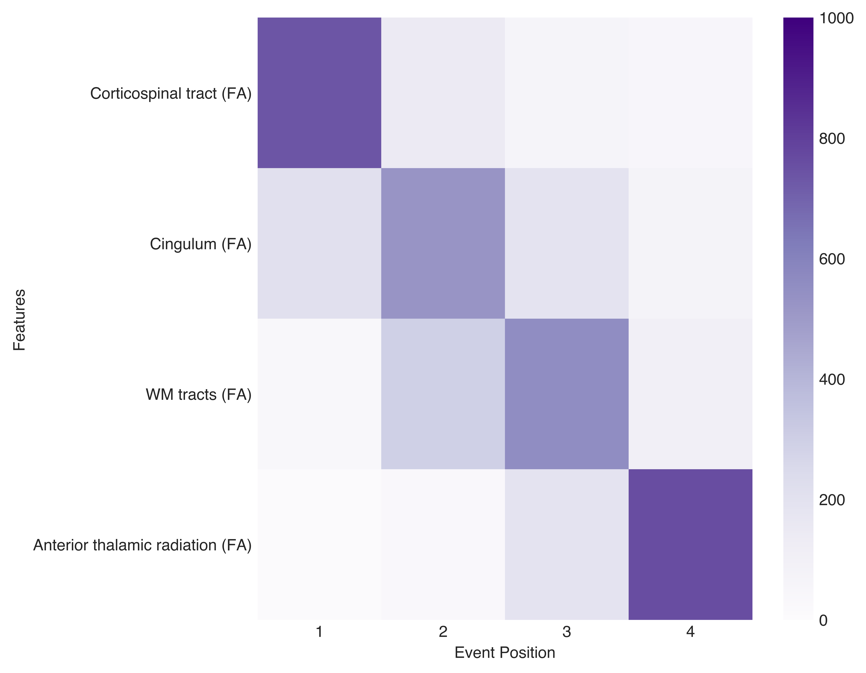


Figure S3: Positional variance diagram for subjects from Model 1 using only the four tract-based biomarkers.

The biomarker ‘WM tracts’ includes several large tracts, which raises the question of their relative positioning to the other three separately included features. In Figure S4 we show the ordering and positional variance of all major tracts in the JHU WM tractography atlas. It can be seen that the relative ordering of the original three separately included tracts from Model 1 does not change when fitting an EBM using all main tracts from the JHU WM tractography atlas (see Figure S4).
The FA of the uncinate fasciculus is positioned after the anterior thalamic radiation at the end of the sequence, and only the inferior longitudinal fasciculus is positioned between the corticospinal tract and the cingulum, albeit with some uncertainty. The other 3 tracts that were previously bundles together as ‘WM tracts’ remain in between the cingulum and the anterior thalamic radiation. Overall, this ordering is well in line with the sequence obtained in Model 1.


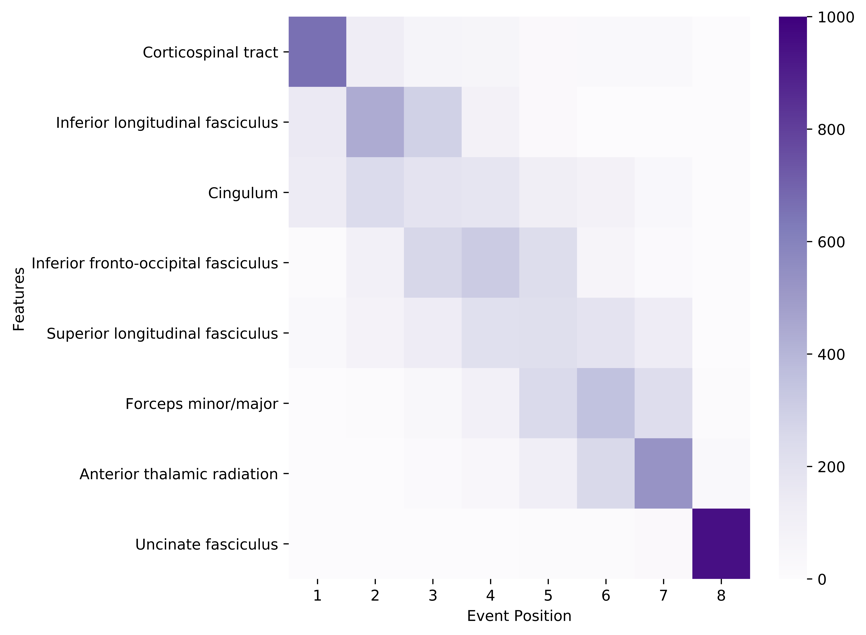


Figure S4: Positional variance diagram for subjects from Model 1 using individual from the JHU atlas instead of merging some of them.
